# Pharmacotherapy of infertility in Ghana: retrospective study at the cape coast teaching hospital

**DOI:** 10.1186/s40545-019-0191-0

**Published:** 2019-11-04

**Authors:** Stephen Mensah Arhin, Kwesi Boadu Mensah, Evans Agbeno, Vitus Sambo Badii, Charles Ansah

**Affiliations:** 1 Department of Pharmacology, Faculty of Pharmacy and Pharmaceutical Science, Kwame Nkrumah University of Science and Technology, Kumasi, Ghana; 2 Department of Obstetrics and Gynaecology, School of Medical Sciences, University of Cape Coast, Cape Coast, Ghana

**Keywords:** Infertility, Prevalence, Treatment, Clomiphene, Africa

## Abstract

**Background:**

Infertility is a major challenge for couples globally. Due to low income levels and the high cost of other assisted reproductive techniques, pharmacotherapy remain the major first line treatment option for infertility in Sub-Saharan Africa.

**Objective:**

The aim of this study was to assess the prevalence of infertility as well as the effectiveness and success achieved following infertility pharmacotherapy at the Cape Coast Teaching Hospital in Ghana.

**Methods:**

This study was a retrospective observational study of 825 couples attending infertility clinic at the hospital.

**Results:**

Prevalence of infertility at the study center was estimated to be 12.3%. Treatment mainly involved the use of clomiphene citrate, antioxidants, herbo-mineral drugs (Ayurveda), multivitamin and antibiotics. Pharmacotherapy resulted in successful conception in one out of every five couples (19.4%; *n* = 160). Secondary infertility, although more prevalent in the study population (44.8%; *n* = 370), had lower conception rates during pharmacotherapy than primary infertility (15% vs 26.2%). Age, kind of infertility, employment status but not educational level were significantly associated with pharmacotherapy success. In ovulation induction, clomiphene citrate plus folic acid and vitamin E adjuncts improved ovulation rates during cycle treatments compared to clomiphene citrate alone. Pharmacotherapy of idiopathic infertility (39%, *n* = 323) was a major challenge with very limited success rates. Interestingly, it was noted that treating couples or female partners only for idiopathic infertility resulted in higher success rates than treating the male partner only. Again, 90-day treatment regimen doubled conception rates when compared with corresponding 30-day treatment regimen. However, zinc sulfate even in short term treatment regimens (30 days) enhanced conception rates in idiopathic infertility.

**Conclusions:**

Prevalence of infertility was estimated to be about 12.3%. One out of every five infertile couples achieved success with pharmacotherapy. Factors such as age, type of infertility, employment status, but not education were significantly associated with treatment success.

## Background

Infertility is one of the main challenges confronting married couples around the world [1–3]. It is defined as difficulty to conceive naturally, following consistent, unprotected intercourse after at least one year [2, 4]. The World Health Organization recognizes infertility as a public health problem [5]. About 10–15% of couples worldwide are unable to achieve spontaneous pregnancy within one year of unprotected intercourse [3, 6, 7]. The proportion of infertile couples in the population varies across the globe but there is a common trend of high prevalence in developing countries [8]and low preponderance in developed countries. In the United States, one out of eight couples who try to conceive for the first time, and one in every six couples who seek a second child are infertile [8, 9] However, in sub-Sahara Africa, prevalence rate vary considerably with some authors reporting rates as high as 21 to 30% in some sub Saharan countries [3, 10]. Estimates in Ghana project infertility as between 11.8–15.8% [11–14]. The notable difference in prevalence between developed and developing countries could be partly due to large disparities in resources for prevention, diagnosis and treatment of infertility [15].

Besides the psychological burden associated with childlessness and the financial implications of treatment, infertility has socio-cultural as well as socio-economic ramifications in many African cultures [2, 3]. Large families were thought to be indicative of wealth, social status and prosperity. In Ghana for instance, “childless” individuals are not considered in any traditional leadership role and are seen as a “disgrace” to their families. Subsequently, existing data about infertility and childlessness in Africa are known to be particularly poor and unreliable as respondents tend to conceal information on child bearing because of being stigmatized [14].

Statistically, various approaches have been employed for estimation of infertility prevalence with each having its peculiar merits and demerits. The Demographic and Health Survey approach assess absence of birth within a five year period. This method, although relevant statistically, has very little clinical relevance for diagnosis and treatment of infertility and it may not provide any information on miscarriages and stillbirths. The Clinical and epidemiological approach addresses this weakness by limiting the duration to 12 to 24 months [16, 17]. It captures couples at risk of becoming pregnant but have delayed conception, who present themselves for evaluation and treatment. The Current duration approach estimates the infertility by the length of time to pregnancy amongst women at risk of pregnancy at the time of the study. It is advantageous because it helps to determine optimal timing of routine infertility and also links population based infertility data to clinical data [16].

Medically, various forms of treatment approaches are employed ranging from pharmacological approaches, to highly sophisticated assisted reproductive techniques. The cost and availability of assisted reproductive techniques such as in-vitro fertilization (IVF), intracytoplasmic sperm injection, surrogacy, cryopreservation, etc. limit the choices of many couples in sub-Saharan Africa to pharmacotherapy [18]. The absence of advanced diagnostic techniques is also a limitation to successful pharmacotherapeutic intervention [19]. Indeed, empirical treatments are mostly employed, especially in cases where no actual cause of the problem can be identified  [20–23].

Although there are international and national guidelines for infertility treatments in sub-Saharan Africa, there is very scanty data on successful treatment outcomes, physician treatment strategies as well as the effect of socio-demographic factors on treatment success. Reviewing and documenting physician treatment strategies, treatment outcomes and the effects of socio-demographic factors on treatment success, can guide and improve infertility treatment in resource constraint settings. To address some of these issues and to provide relevant scientific data and clinical insight, this study was carried-out in a major fertility Clinic in Ghana, West Africa.

## Methods

### Study area

The study was conducted at the Cape Coast Teaching Hospital in the Central Region of Ghana (CCTH). The Cape Coast Teaching Hospital is currently a 400 bed capacity referral Hospital situated at the Northern part of Cape Coast. It is bounded on the north by Abura Township, on the south by Pedu Estate / 4th Ridge, Nkanfua on the East and Abura / Pedu Estate on the West. It serves as the referral point for the people within the entire Cape Coast Metropolis, Central Region as a whole and even beyond. The hospital has specialist gynaecologists who see higher number of infertility cases than any other health facility in the region.

### Study design

This was a five-year retrospective study in which data was collected from patient medical records filed in the hospital. The primary target were couples with infertility problems who attended fertility clinic from January 2011 to December 2015.

### Sample size

The estimation of 300 patients was based on average hospital attendance for infertile couples per year using the formula modified by Naing et al.*,* [24] as shown $\frac{n={Z}^2x\ (p)\ x\ \left(1-p\right)}{e^2}$

Where Z = 1.96 (z value 1.96 of 95% confidence level).

e = 0.05 (Margin of error).

*p* = 0.3 (arbitrary proportion of patients with infertility).

### Sampling method

Folder numbers of all patients who presented with various kinds of infertility problems over the five-year period, from 2011 to 2015 were collected from the Out Patients Department (OPD) consultation room book where fertility clinic was conducted. A total of about 4000 folders were available for randomization. A systematic random sampling technique was used to select respondents. Out of the total number of patients seen during the various years under study, a constant number (K^th^) was determined, such that each (3rd) patient medical record in respect of each year was selected and reviewed. Three hundred folders were selected from each year of the study by means of systematic random sampling, making a total of 1500 folders. The records unit was able to extract only 1350 folders. This was done with the help of computer generated codes, after the folder numbers were keyed into the programmed computer at the hospital database. The folders were thoroughly examined using the inclusion and exclusion criteria. Out of the 1350 folders that were retrieved, only 825 patients met the selection criteria. Data for hospital attendance with age categories for the entire five years was obtained from the hospital statistical unit.

The inclusion criteria were; patients with infertility for at least one year, and couples with complete medical history and have gone through all basic necessary investigations. Excluded from the study were patients with infertility of less than one year duration, patients with co-morbid conditions such as, retroviral infection, hepatitis B infection, thyroid disorders, cancer of the cervix, endometrium, colon, brain and pituitary carcinomas, uterine fibroids, tubal blockages and incomplete medical history.

### Characteristics of patients and outcome measure/definition

The general classification of the patients to primary infertility, secondary infertility or subfertility were sorted as recorded in the out patients’ consultation book. Primary infertility was defined as a person who has never had a live birth due to failure to become pregnant or lack of capacity to carry pregnancy to a live birth. Secondary infertility described persons who had previously conceived, resulting in live birth, but finds it difficult to conceive again and achieve live birth. Subfertility was defined as the time of undesirable non-conception that the couples experience, with higher probability of getting pregnant without necessarily undergoing treatment. These patients were identified when couples report unwanted/delayed intervals of conception. The main primary outcome of the study was occurrence of ovulation and clinical pregnancy. The outcome criteria were determined by measurement of day 21 progesterone level to determine ovulation. Urine pregnancy tests were done to confirm the presence of pregnancy after the women have reported missing the period at the expected dates.

### Treatment of patients

Various laboratory investigations, appropriate to sex were performed before treatments were initiated. Medications were written to patients based on the cause of their problem, whereas empirical treatments were given to couples with unknown etiologies. Treatment of women with clomiphene citrate begun either on the 3rd, 4th, or 5th day following natural menstrual flow or progesterone withdrawal tests. Those treated with adjunct therapies began same day with clomiphene. Adjunct therapies were either prescribed or not for couples seeking fertility treatment, depending on the prescriber’s decision. The clomiphene citrate dosages were held constant for a particular cycle, either alone or with varying forms of adjunct therapies.

### Estimation of infertility prevalence

The clinical estimation of infertility prevalence was done based on WHO formula for Reproductive Health Indictors [25] as shown below:

Prevalence (P).

P = Total No. of patients in their reproductive age (15–49) yrs., at risk of conceiving but unable to conceive for at least 1 yr. × 100 **/** total No. of patients in their reproductive age who reported to the hospital within the study period aged (15–49) yrs. at risk of becoming pregnant.

### Statistical analysis

Data obtained were captured in a data capture form for easy analysis and evaluation. Data was analyzed using SPSS version 20, Microsoft Excel Spreadsheet, and Graph Pad Prism version 16. Categorical variables were analyzed using chi-square test, one-way and two-way analysis of variance was used to analyze difference in mean values between groups. A 95% confidence interval was selected such that *p*-value less than 0.05 were considered significant. Dunnett’s multiple comparisons post hoc test was used.

## Results

### Respondents’ infertility characterization

**Fig. 1:**
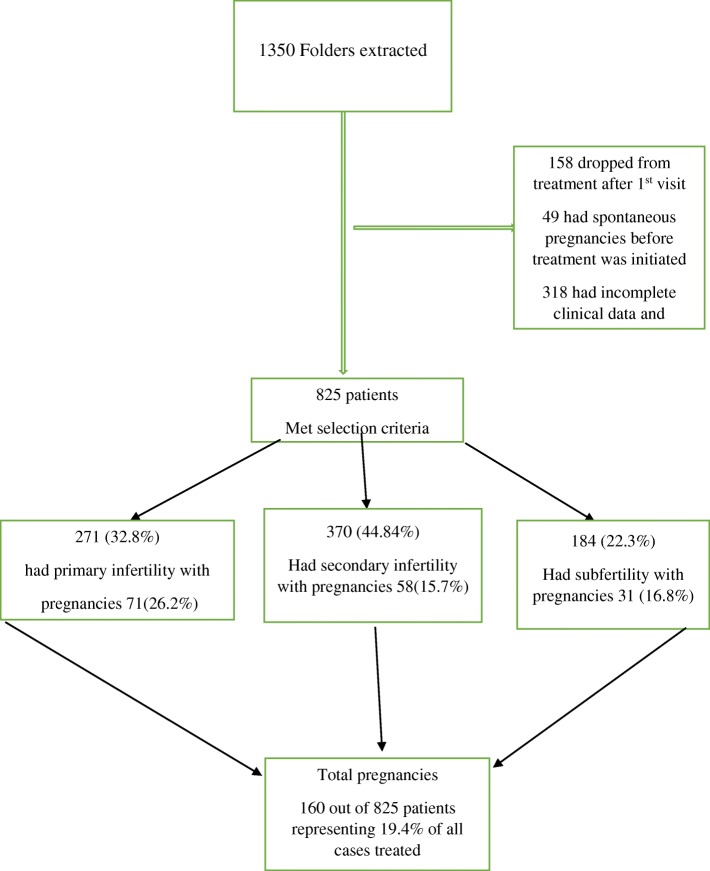
Diagram showing a summary of patient sampling, diagnosis and pregnancy outcome

A total of 825 couples met the selection criteria (Fig. 1). The minimum age for the female partners was 18 years and maximum age of 45 years. Out of the total number that met inclusion criteria, 44.8% (*n* = 370) of couples had secondary infertility, 33% (*n* = 271) had primary infertility and 22.3% (*n* = 184) had subfertility. Couples in the primary infertility category were significantly younger and had experienced few years of infertility than those in the secondary and subfertility category. There was no difference in age between couples in the secondary or subfertility category. However, couples classified as sub-fertile had experienced infertility for a significantly longer duration than secondary couples (Table 1). For respondents with little or no education, subfertility was predominant compared with primary infertility which appeared to be predominant amongst respondents with a tertiary education. Secondary infertility was even across all educational groups.

**Table 1:** Socio-Demographics of infertility amongst respondents

DEMOGRAPHICS OF INFERTILITY AMONGSTS RESPONDENTS				
Type of infertility		Primary (*N* = 271)	Secondary (*N* = 370)	Sub fertility (*N* = 184)
Infertility Class (%)		33	44.8	22.3
Mean Age (years)		25.89 ± 4.1	29.58 ± 4.6^a^	30.44 ± 3.23^a^
Duration of Infertility (years)		3.36 ± 1.70	5.70 ± 2.11^ab^	6.41 ± 2.65 ^ab^
Occupation (%)	Employed(*n* = 564)	31.2	45.9	22.3
	Unemployed(*n* = 213)	39.9	40.4	19.7
Education (%)	Primary/no-education (*n* = 267)	29.6	44.9	25.5
	Secondary (*n* = 276)	31.7	34.3	34.2
	Tertiary (*n* = 247)	35.4	30.8	25.0
Religion (%)	Christian (*n* = 603)	32.7	43.1	24.2
	Muslim (*n* = 109)	34	45.8	19.2
	Others (*n* = 22)	54.5	26	0.09

Results presented as percentages. Age and duration of infertility present as Mean Mean ± SD. Statistical analysis of Age and duration of infertility is by One-way analysis of variance followed by Bonferoni test Post Hoc Test. (a) means significantly different from the Primary infertility, (b) means significantly different from secondary infertility. *P* < 0.001

### Infertility prevalence

The method for the estimation of infertility prevalence in the current study was based on the clinical approach model. From the OPD consultation book, all couples at risk of pregnancy who reported trying to conceive for at least one year at the fertility clinic within the five-year period was =3682. Total hospital attendance for the hospital within the five-year period for patients within the age range (15–49) yrs. and at risk of pregnancy as measured against the women’s age, was about 29,891. Hence using the formula at the method section of the study, prevalence was estimated to be 12.3%.

### Causes and sex specific diagnosis of infertility

Approximately 39% (*n* = 323) of couples had infertility of no identifiable or unexplained cause (idiopathic). Infections were identified as the cause of 21.3% (*n* = 175) of infertility in the study. Over 26% (*n* = 215) of infertility was attributed to abnormality in the ovulatory process (Table 2). Of the 215 respondents with ovulatory problems, one half (50%) were diagnosed as oligo-ovulatory and 38% of the people were anovulatory. One in every six women, thus 12% (*n* = 26) with ovulatory problems, suffered from polycystic ovary syndrome. Sperm anomalies only accounted for 13.6% (*n* = 112) of infertility of which 58% (*n* = 65) were identified as oligozoospermia. Thirty percent (30%) of men with sperm anomalies were diagnosed as Oligoasthenozoospermia. One in every six did not produce sperm cells at all (azoospermic) (Table 2).

**Table 2:** Causes of infertility, Sex Specific diagnosis and treatment Success

Specific causes of couple infertility and treatment Success						
Type of infertility	Causes of infertility n (%)				Treatment success	
	Idiopathic	Sperm anomalies	Ovulation dysfunction	Infections	Conception rate (%)	*P* value
Primary (*N* = 271)	112 (41.3)	32 (11.8)	87 (32.1)	40 (14.8)	71 (26.2)	*P* = 0.002
Secondary(*N* = 370)	132 (35.7)	59 (15.9)	75 (20.3)	104 (28.9)	58 (15.7)	
Subfertility(*N* = 184)	79 (42.9)	21 (11.4)	53 (28.9)	31 (16.8)	31 (16.8)	
Total no of couples (*N* = 825)	**323 (39.2)**	**112 (13.6)**	**215 (26.1)**	**175 (21.1)**	**160 (19.4)**	
Sex specific diagnoses of infertility n (%)						
OVULATION DYSFUNCTION (*n* = 215)	Anovulation	82 (38.4)				
	Oligo-ovulation	107 (49.8)				
	PCOS	26 (12.1)				
SPERM ANOMALIES (*n* = 112)	Oligozoospermia	65 (58.0)				
	Oligoasthenozoospermia	34 (30.4)				
	Azoospermia	13 (11.6)				

### Biodata of patients and their impact on treatment outcome

In general, primary infertility had higher treatment success rates compared with secondary infertility and subfertility (Table 2). Age was a very significant factor in treatment success as younger couples had higher chance of conception than older couples (Table 3). Again, the longer the duration of infertility, the lower the rate of conception. Education did not affect the chances of conceiving but the employment status significantly affected the success of conception (*P* = 0.005). Religion had no effect on conception.

**Table 3:** EFFECTS OF SOCIODEMOGRAPHICS ON THE SUCCESS OF INFERTILITY TREATMENT

SOCIO DEMOGRAPHY AND INFERTILITY TREATMENT SUCCESS				
DEMOGRAPHIC FACTORS		CONCEPTION RATE (%)		
		Yes	No	Sign
AGE	< 30	91(24.1)	286 (75.9)	*P* = 0.001 X^2^ = 18.2
	30–35	53(18.2)	238(81.8)	
	36>	16(10.2)	157(89.8)	
DURATION OF INFERTILITY	1–5	98(24.4)	304(75.6)	P = 0.002 X^2^ = 12.9
	5–10	51(15.4)	281(84.6)	
	10>	11(12.5)	80(87.9)	
EDUCATION	Primary	33(18.2)	148 (81.8)	*P* = 0.47 X^2^ = 2.50
	Secondary	50(18.3)	223(81.7)	
	Tertiary	56(22.8)	190(77.2)	
	No Formal	21(23.1)	70(76.9)	
OCCUPATION	Employed	102(18.1)	463(81.9)	P = 0.005 X^2^ = 7.9
	Unemployed	58(27.2)	155(72.8)	
RELIGION	Christian	125(20.7)	478(79.3)	*P* = 0.197 X^2^ = 3.25
	Islamic	27(24.8)	82(75.2)	
	Others	8(34.8)	15(65.2)	

Cross tabulation of demographic factors against pregnancy outcome using chi-square test

### Pharmacotherapy of infertility

Several classes of medications were used to treat couples with fertility problems who attended the fertility clinic. A quarter of the respondents (*n* = 200) were administered vitamin E for infertility. A fifth of all respondents were prescribed folic acid in addition to other medicines i.e. 20% (*n* = 166). Twelve percent of respondents (*n* = 99) were either on Clomiphene Citrate (mostly women) and Ayurveda drugs (mostly men). Each cycle of clomiphene had the same dosage with varying amounts of adjunct therapies. Medroxyprogesterone acetate was commonly prescribed to women for the progestin withdrawal test. In general, male partners with sperm anomaly after the seminogram test were treated with Ayurveda drugs and antioxidant complexes whilst females were usually treated with clomiphene citrate with or without folic acid, multivitamin adjuvants. Vitamin E, zinc sulphate and antibiotics were commonly used in both sexes as well.

### Effects of adjunct therapies on efficacy of clomiphene citrate

**Fig. 2:**
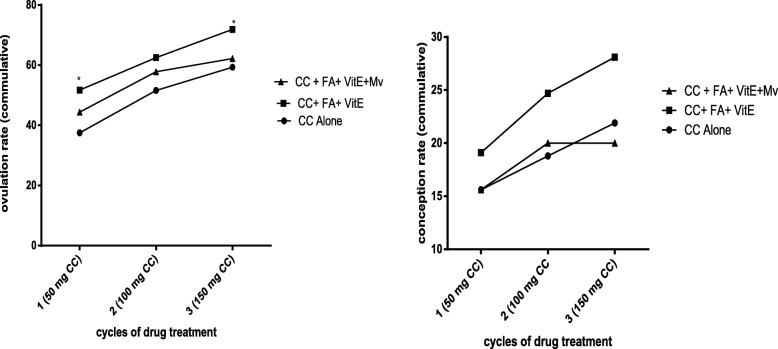
Effects of Adjunct therapy of folic acid (5 mg) + vitamin E (400 IU) with or without multivitamin (one tablet daily) on cycles of Clomiphene citrate for inducing ovulation and conception in women with ovulatory problems. Each cycle had the same dosages of clomiphene but varying forms of adjunct therapies. Clomiphene citrate was used for five days. Both the clomiphene and adjunct therapies were started same day but the adjuncts were extended to 30 days. Statistical analyses by 2-way ANOVA using Dunnett multiple comparison. * means *p* < 0.05

In the study, it was noted that ovulation rates and conception rates improved with the cycles of treatment with clomiphene citrate (i.e. 50 mg, 100 mg, 150 mg) (Fig 2). Generally, addition of Folic acid +Vitamin E with or without multivitamin to clomiphene resulted in higher ovulations and conceptions compared to clomiphene alone. Thus, the cyclical use of clomiphene citrate alone (*n* = 64) resulted in ovulation rates of 24 (37.5%), 33(51.55), 38 (59.4%) and conception rates of 10 (15.6%), 12 (18.8%), 14 (21.8%) at the (50 mg, 100 mg and 150 mg) doses respectively. However, the addition of FA+ Vit E (*n* = 89) resulted in higher ovulation rates of  46 (51.35), 58 (65.2%), 64 (71.9%) and conception rates of 17 (19.1%), 22 (24.7%), 25 (28.1%) at all the doses of CC. Similarly, addition of FA+ Vit E+ Mv (*n* = 45) resulted in ovulation rates of 20 (44.4%), 26 (57.7%), 28(62.2%) at all CC cycle respectively and conception rates of 7 (15.6%), 9(20.0%) at 50 and 100 mg dose respectively. The 150 mg dose of CC with FA + Vit E + Mv did not result in conception. Overall, the addition of adjuncts (FA + Vit E) to CC cycles resulted in statistically significant improvement in ovulation rates. Although conception rates improved as well, it was not statistically significant (Fig. 2).

### Treatment approaches for idiopathic/unexplained infertility

Idiopathic infertility presented a major challenge to the clinic. From the study, it was noted that treating couples or the woman alone presented higher conception rates than treating the man alone although this was not statistically different (*p* = 0.07). Again, duration of treatment also affected conception rate. Couples treated for 90 days had almost double the conception rate than couples given similar regimen for 30 days. Addition of zinc sulfate also increased conception rate. Interestingly, addition of zinc almost triple conception rate of folic acid plus Vitamin E combination for women even if the duration of treatment was only 30 days. Using clomiphene citrate for the women always resulted in higher conceptions compared with a combination of Vitamin E and Folic acid. (Table 4).

**Table 4:** Pharmacotherapy for idiopathic Infertility, M = Male, F=Female

Pharmacotherapy of Idiopathic infertility		
	Treatment approach/Strategy	Conception Rates n (%)
Treatment Partner	Males only (*n* = 64)	8 (12.5)
	Females only (*n* = 106)	21 (19.4)
	Couple (*n* = 133)	26 (19.5)
Duration of Treatment	Treatment for 30 days	7.20 ± 2.33
	Treatment for 90 days	**13.33 ± 1.93**
Couple Treatment Regimen	Antioxidant Complex X 30 (M) FA + VitE X 30 (F) (*n* = 20)	2 (10.0)
	Antioxidant Complex X 90 (M) Female FA + VitE X 90 (F) (*n* = 12)	2 (16.7)
	Antioxidant Complex X 30 (M) Clomiphene Citrate 50 mg X 30 (F) (*n* = 32)	6 (18.8)
	Ayurveda (adezoa) X 90 (M) FA + VitE X 90 (F) (*n* = 25)	4 (16.0)
	Ayurveda (adezoa) X 90 (M) (*n* = 44) Clomiphene Citrate 50 mg X 90 (F)	12 (27.3)
	FA + Vit E + Zn X30 (Only F) (*n* = 17) FA + Vit E X30 (Only F) (*n* = 24)	2 (11.8) 1 (4.2)

M and F represents male and female respective treatment regimen

## Discussion

Global trends depict Sub-Saharan Africa as experiencing a “baby boom” in the twenty-first Century compared with many other regions of the world [26]. Although perceptions seem to be gradually eroding, many African cultures still see large family as indicative of wealth and social status [2, 3]. However within this apparent boom are couples struggling with infertility. Sociocultural stigmatization drive many couples to seek medical help. Because of low income levels, pharmacotherapy remains the main treatment option for infertility. Data on infertility rates and the success of pharmacotherapy remains very scanty. The study therefore assessed the prevalence of infertility and pharmacotherapy of infertility at the Cape Coast Teaching Hospital in Ghana.

In this study, the prevalence of infertility was found to be 12.3% at the study center; this was consistent with estimates by Boivin *et al*., in 2007 and Mascarenhas *et al*., in 2012, although the methods used in this current study differed significantly. Secondary infertility was the most common amongst respondents. However, pharmacotherapy resulted in higher conception rates in respondents with primary infertility. This may not be surprising since demographically, primary infertility was predominantly found in young couples. This also confirms research reported elsewhere by other authors [14, 27]. The mean age and duration of infertility of couples with primary infertility was significantly lower and may have contribute to the higher conception rates. This difference in age and duration presupposes that early detection and treatment of other forms of infertility may impact positively on outcomes. However, because couples with primary infertility have never achieved a live birth during the course of their union and hence more likely to be stigmatized [2, 3], there may be an additional motivation to seek prompt medical help and to rigorously adhere to fertility treatment [28]. The high success noted in this study can be a guide to counselors in re-assuring couples with primary infertility of a high probability of success which will have a psychological benefit of reducing unnecessary anxiety during treatment. Clinicians managing sub fertile couples and secondary infertile couples need to develop strategies that will motivate their clients to adhere to treatment. Again, fertility counselors should be aware and be able to manage the “better than average/ illusionary superiority effect” that sub fertile couples and secondary infertile couples may have by virtue of a previously successful live birth.

The study recorded an unusually higher proportion of infertility of idiopathic etiology (unexplained infertility) compared with findings from other studies [29–31]. As to whether our results is a true representation of infertility in Ghana, or confounded by a misdiagnosis by Clinicians, partly because of limited resources in developing countries, as reported in other studies, remains to be determined. The poor success rates achieved during pharmacotherapy of unexplained infertility may suggest a probable misdiagnosis of the condition.

In our study of couple infertility, female associated infertility factors were two times higher than male associated infertility factors. This is in contrast to the work by Ikechebelu *et al.,* 2003 who reported that male associated infertility as the leading cause of couple infertility in Africa. However, both studies show clearly that infertility could originate from either partners and hence a couple directed treatment may be the best treatment strategy. Pelvic infections were observed to be one of the major causes of infertility among couples and this was in line with other works [27, 31, 32]. Policy that aims at intensifying campaign against sexually transmitted infections (STI_S_) and early diagnosis and treatment of sexually transmitted infections will be a preventive measure in reducing infertility in sub-Saharan Africa. Furthermore enforcing national regulations on antibiotic therapy and avoiding the use of substandard, counterfeit antibiotics can reduce the incidence of drug-resistant microbes that cause persistent STIs [33, 34].

Most of the prescribed pharmacologic agents such as Vitamin E, Folic acid, ayurveda, and antioxidant complexes work by reducing the deleterious effects of reactive oxygen species [35, 36]. These agents were usually prescribed for couples with sperm anomalies and infertility of idiopathic etiology, in which oxidative stress may be major contributing factor [37, 38]. Women with ovulation dysfunction were mostly prescribed clomiphene citrate in combination with adjuvants such as Vitamin E, folic acid, and sometimes with or without multivitamin. These combinations resulted in higher conception rates compared to clomiphene citrate alone. There is substantial clinical evidence demonstrating clinical efficacy of clomiphene, vitamins, and antioxidant combination therapies in male and female reproduction [39–41]. The reason could be the capacity of the adjuvant therapies to improve blood flow and quality of germ cells through their free radical scavenging activity [39–41]. It is very possible that some prescribers did not recommend the use of adjunct therapy not because of misinformation on their clinical efficacy but rather to minimize the financial burden on their clients. Availability of cheaper and yet efficacious alternatives such as folic acid, zinc sulfate noted in this study may promote their use.

Prescriptions for the treatment of couples with unexplained infertility did not follow any standard protocol, as different prescribers used different drug combinations, thereby confirming the empiricism in treatment [19, 22, 40]. Though the treatment of unexplained infertility generally presented with low pregnancy rates, couples treated for longer durations always did better independent on the kind of pharmacological agent used. Interestingly, the addition of zinc sulfate seem to potentiate the efficacy of clomiphene citrate and folic acid and vitamin E combinations independent on the duration of treatment. However, authors could not make an extensive study of this effect because of the limited number of patients on these drug combination subgroup.

To the best of our knowledge, this study is the first in its kind to assess the outcome of pharmacotherapeutic management of infertility in Ghana. This study has provided relevant information on effects of several medicines that are widely used for pharmacotherapy in sub-Saharan Africa as well as the importance of adjunct therapy. The study identified several demographic factors that affected pharmacotherapy of infertility. From the study, there is need for greater research into the study of unexplained/ idiopathic infertility.

### Limitations of the study

One of the main limitations was the retrospective nature of the study. It was not possible to access the psychological status of the patients who could not conceive after the series of pharmacological treatment. Also the outcome of live births could not be evaluated due to lack of follow up. We could not also look into the in-depth analysis of various laboratory investigations though it played a crucial role in arriving at the final diagnosis. Moreover, because patients were prescribed different drug combinations, an extensive comparison between all the various combinations could not be performed. In addition, the retrospective nature of the study as well as the small sample size when the groups are divided into adjuvant treatment groups was also a major limitation. The strength of the study, however, was that comparisons were done for the different treatment combinations that were used for the same durations, and that could serve as a source of direction for clinicians to know which combination of drugs are effective for the treatment of the couples.

## Conclusions

The prevalence of infertility was estimated to be 12.3% at the study center. However, the rates of secondary infertility was higher than primary infertility and subfertility. The addition of the adjuvant therapies had shown to improve ovulatory and conception rates. Longer treatment duration was associated with higher pregnancy rates especially in couples with idiopathic infertility. Alternative combination therapy involving zinc sulfate may be very effective in short term treatments.

## Data Availability

All data relevant to the work has been provided.

## Abbreviations

CC: Clomiphene citrate
CCTH: Cape Coast teaching hospital
CD: Current duration
DHS: Demographic health survey
F: Females
FA: Folic acid
IVF: In-vitro fertilization
M: Males
Mv: Multivitamin
Vit E: Vitamin E
Zn: Zinc
